# Estrogens Correlate with *PELP1* Expression in ER Positive Breast Cancer

**DOI:** 10.1371/journal.pone.0134351

**Published:** 2015-08-06

**Authors:** Marianne Hauglid Flågeng, Stian Knappskog, Jennifer Gjerde, Per Eystein Lønning, Gunnar Mellgren

**Affiliations:** 1 Hormone Laboratory, Haukeland University Hospital, Bergen, Norway; 2 Department of Clinical Science, University of Bergen, Bergen, Norway; 3 Department of Oncology, Haukeland University Hospital, Bergen, Norway; Roswell Park Cancer Institute, UNITED STATES

## Abstract

The Proline-, glutamic acid- and leucine-rich protein 1 (*PELP1*) is an estrogen receptor (ER) coactivator and a proto-oncogene known to be deregulated in endocrine cancers. In breast cancer, *PELP1* overexpression has been associated with endocrine therapy resistance. Although *PELP1* is known to be regulated by estrogens *in vitro*, its association with estrogen levels within the tissue of breast cancer patients has not previously been assessed. Here, we determined *PELP1* mRNA expression levels in paired samples of normal and malignant breast tissue obtained from 32 postmenopausal and 11 premenopausal women. In the total sample set, *PELP1* levels were higher in tumors compared to normal breast tissue (*P* = 0.041). Among postmenopausal women, *PELP1* tumor levels correlated positively with estrone (E_1_) and estradiol (E_2_) levels in both normal tissue (r = 0.543, *P* = 0.003 and r = 0.601, *P* = 0.001, respectively) and plasma (r = 0.392, *P* = 0.053 and r = 0.403, *P* = 0.046, respectively). Analyzing all ER+ tumors (n = 26), *PELP1* correlated positively with E_1_ and E_2_ in tumor tissue (r = 0.562, *P* = 0.003 and r = 0.411, *P* = 0.037, respectively) and normal tissue (r = 0.461, *P* = 0.018 and r = 0.427, *P* = 0.030, respectively) in addition to plasma E_1_, E_2_ and estrone sulphate (E_1_S) concentrations (r = 0.576, *P* = 0.003, r = 0.456, *P* = 0.025 and r = 0.406, *P* = 0.049, respectively). Finally, *PELP1* correlated positively with ER mRNA (*ESR1)* (r = 0.553, *P* = 0.026) in ER+ tumors, whereas a negative association between *PELP1* and *ESR1* (r = -0.733, *P* = 0.010) was observed in ER- breast tumors. Taken together, tumor *PELP1* mRNA expression is associated with estrogen levels in breast cancer, suggesting a potentially important role of *PELP1* in ER+ breast cancer growth *in vivo*.

## Introduction

The human estrogen receptor (ER) plays an important role in development and progression of breast cancer. Elevated estrogen levels have been associated with higher risk of incident cancer in postmenopausal women (summarized in [1]). Approximately 75% of all breast cancers are ER-positive, thus blocking the growth stimulatory effects of estrogens by endocrine therapy is a major treatment option in breast cancer.

The transcriptional capacity of ER is highly dependent on coregulators (coactivators and corepressors) which regulate its transcriptional activity (reviewed in [2]). ER coregulators have been shown to play a role in endocrine responsiveness and development of resistance to endocrine treatment [3–5]. The coactivator proline-, glutamic acid-, and leucine-rich protein (PELP) 1 is an ER coactivator and proto-oncogene, which is dysregulated in breast cancer and associated with poor survival [6–9]. PELP1 is overexpressed in 60–80% of breast tumors [6–9] and plays important roles in both ER genomic and non-genomic signaling [10, 11]. In the nucleus, PELP1 interacts with a number of transcription factors [10]. The proto-oncogenic functions of PELP1 involve different cellular processes including epigenetic modifications leading to ER transactivation and breast cancer progression [12–14]. Furthermore, PELP1 activates kinase cascades in the cytoplasm such as MAPK activation via c-Src and PI3K signaling [9, 11, 15, 16].

PELP1 expression is upregulated by ER in breast cancer cells *in vitro* [17]. Recently, it was also demonstrated that overexpression of PELP1 in murine mammary glands resulted in development of hyperplasia and carcinoma [18]. However, the potential role of PELP1 as an executor of estrogens pro-carcinogenic effects in human breast cancer remains to be verified.

In the present study, we explored potential associations between PELP1 and E_2_-dependent ER signaling in breast cancer patients. We analyzed PELP1 mRNA expression levels in breast cancer and normal tissue samples and potential connections to ER- and postmenopausal status of the patients. Moreover, we correlated *PELP1* with the ER mRNA expression (*ESR1*) and estrogen levels in plasma, normal breast tissue and tumor tissue [19]. Our findings add novel information regarding association and, potentially, regulation of the oncogene *PELP1* by estrogens in ER+ breast cancer.

## Materials and Methods

### Study population and sample collection

The study population has been described in detail elsewhere [19]. A total of 13 premenopausal and 34 postmenopausal patients selected for mastectomy at the Department of Surgery, Haukeland University Hospital, Bergen, Norway, were enrolled. Tissues obtained from mastectomy specimens, both normal and tumor tissue, were removed and immediately snap-frozen in liquid nitrogen in the operating theatre, before they were stored in liquid nitrogen until use. Normal tissue was isolated from the breast quadrant farthest from the tumor-containing quadrant in the breast. Blood samples for plasma measurements were obtained at the day of surgery after fasting overnight, and stored at -20°C until use. Both tumor and normal breast tissue were not available for gene expression analysis from one patient in addition to normal breast tissue from one patient and tumor tissues from two further patients, leaving 43 patients for statistical comparisons between tumor and normal tissue. Additionally, four of the patients had received hormone replacement therapy within the 4 weeks pre-surgical period and were excluded from the correlation analysis with the estrogen levels.

### Ethics statements

The protocol for the study was presented and exempted from review by the Regional Committee for Medical and Health Research Ethics (REK) at the time of collection. The study was performed in accordance to Norwegian law and regulations, and all patients provided written informed consent. After the samples had been collected, each patient was allocated a trial number, demographic data was collected, and the database anonymized.

### Real time PCR quantification

Total RNA was extracted from ~25 mg tissue using Trizol (Invitrogen, Carlsbad, CA) according to the manufacturer’s recommendations. RNA concentrations were estimated by optical density (OD) measurement using the Nanodrop (Saveen Werner, Copenhagen, Denmark). For each sample, 1 μg total RNA was reversely transcribed by the 1^st^ Strand cDNA Synthesis Kit (Roche, Basel, Switzerland) using random primers. The cDNA was diluted 1/10 in PCR-grade water and stored at -20°C until use.

Real time PCR analyses were performed in three parallel runs on a Light Cycler 480 (LC480) Thermo Cycler (Roche, Basel, Switzerland). A negative control was included in each run. Three reference genes were analyzed in each sample: Gene-specific primers and probes for PELP1 and the reference genes Pumilio homolog 1 (PUM1) and Ribosomal protein, large, P0 (RPLP0) were designed using Universal Probe Library (UPL, Roche, Basel, Switzerland, S1 Table). Reference analysis kit (Roche, Basel, Switzerland) was used for the TATA-box binding protein (TBP) reference gene. Amplification reaction mixture consisted of 2.5 μL diluted cDNA, 10 μL LC480 Probe Master mix (Roche, Basel, Switzerland), 0.4 μmol/L of each primer, 0.2 μmol/L of UPL probe, or 0.2 μmol/L of TBP reference primers and 0.1 μmol/L TBP reference probe in a total volume of 20 μL. Thermocycling setup used was as following: pre-incubation at 95°C for 10 minutes, 45 cycles with denaturation at 95°C for 10 seconds, primer annealing at 60°C for 30 seconds and DNA sequence extension at 72°C for 1 second followed by fluorescence measurement. The PCR products were then cooled at 40°C. Crossing points (Cp) and the standard curve efficiency from a serially diluted cDNA sample were used to quantify relative expression levels of each target gene separately. *PELP1* mRNA was detected well in both tissues, with an average Cp of 29.9 (range: 26.1–37.8) in the total data set. Data are presented relative to the mean value of the three reference genes in each single sample.

*ESR1*, *HER1* and *NRG1* mRNA expression levels in tumors have been analyzed and reported previously [20, 21].

### Measurement of estrogen levels

Estrogen levels measured in plasma and the paired normal and tumor tissue samples from 13 premenopausal and 30 postmenopausal women have previously been reported [19]. In brief, estrogen fractions were measured with highly sensitive RIA methods, subsequent to pre-analytical purification through LH20 column (plasma) or HPLC (tissue) chromatography [22, 23]. Sensitivity limits for the different analysis were 19.8 fmol/g for estrone (E_1_), 4.3 fmol/g for estradiol (E_2_) and 11.9 fmol/g for estrone sulphate (E_1_S) in tissue, whereas in serum the sensitivity limits were 1.14 pmol/L for E_1_, 0.67 pmol/L for E_2_, and 0.55 pmol/L for E_1_S [22, 23].

### Statistical analysis

The mRNA expression level is presented as geometric mean with 95% confidence interval (CI) of the mean. We used Spearman Rank test to perform correlation analyses of *PELP1* levels in normal and tumor tissue with levels of E_1_, E_2_ and E_1_S in normal tissue, tumor tissue and plasma. Additionally, the same test was used to analyze correlations between *PELP1* and *HER1* and *NRG1* during estrogen deprivation. A multivariate binary regression analysis was performed to analyze association between the covariates *PELP1* and *ESR1* dependent of ER status. Differences in mRNA expression between paired tumor- and normal-tissue samples were analyzed using the non-parametric Wilcoxon signed rank test. Differences in *PELP1* levels between ER+ and ER- or pre- and postmenopausal subjects were analyzed using non-parametric Mann-Whitney U rank test of independent samples. All *P*-values were two-sided and the threshold *P*-value for statistical significance was 0.05. All analyses were performed using the software SPSS Statistics version 19 (IBM SPSS Statistics).

## Results

### Patient characteristics and tissue specimens

The study population includes 47 pre-and postmenopausal breast cancer patients with ER+ and ER- disease (Table 1), described in detail elsewhere [19]. Among these, 43 patients, 32 postmenopausal and 11 premenopausal, were available for gene expression analyses both from normal and malignant breast tissue.

**Table 1:** Patient, tumor and treatment characteristics.

		ER-	ER+
Breast cancer patients (n = 47)			
Pre- or postmenopausal^a^	Pre	6	7
	Post	9	25
HRT	No	15	28
	Yes	0	4^b^

Abbreviations: ER, estrogen receptor; HRT, hormone replacement therapy

^a^ Both normal breast and cancer tissue were available for gene expression analyses from 11 premenopausal and 32 postmenopausal patients.

^b^ These four patients were excluded from the correlation analysis with the estrogen levels.

### Expression of PELP1 mRNA in normal and malignant breast tissue

The ER-coactivator *PELP1* was well detected in both normal and malignant breast tissue and its mRNA expression level is presented relative to the mean of the three reference genes *TBP*, *PUM1* and *RPLP0*. Comparing normal and malignant breast tissue for each individual patient in the total data set we observed elevated *PELP1* levels in 25 out of 43 tumors. In malignant breast tissue geometric (geo) mean of *PELP1* expression was 1.37 (95% Confidence Interval (CI): 1.22–1.53), compared to normal tissue with geo mean of 1.25 (95% CI: 1.15–1.37, *P* = 0.041, S1 Fig). No significant differences were observed in tumor *PELP1* levels between the subgroups of pre- and postmenopausal (geo mean: 1.41 95% CI: 1.10–1.81; 1.35, 95% CI: 1.19–1.52, respectively) or ER+ and ER- subjects (geo mean: 1.47, 95% CI: 1.11–1.94 and 1.33, 95% CI: 1.18–1.50, respectively, S2 Fig).

### Correlations between tumor PELP1 mRNA and estrogen levels

**Fig 1:**
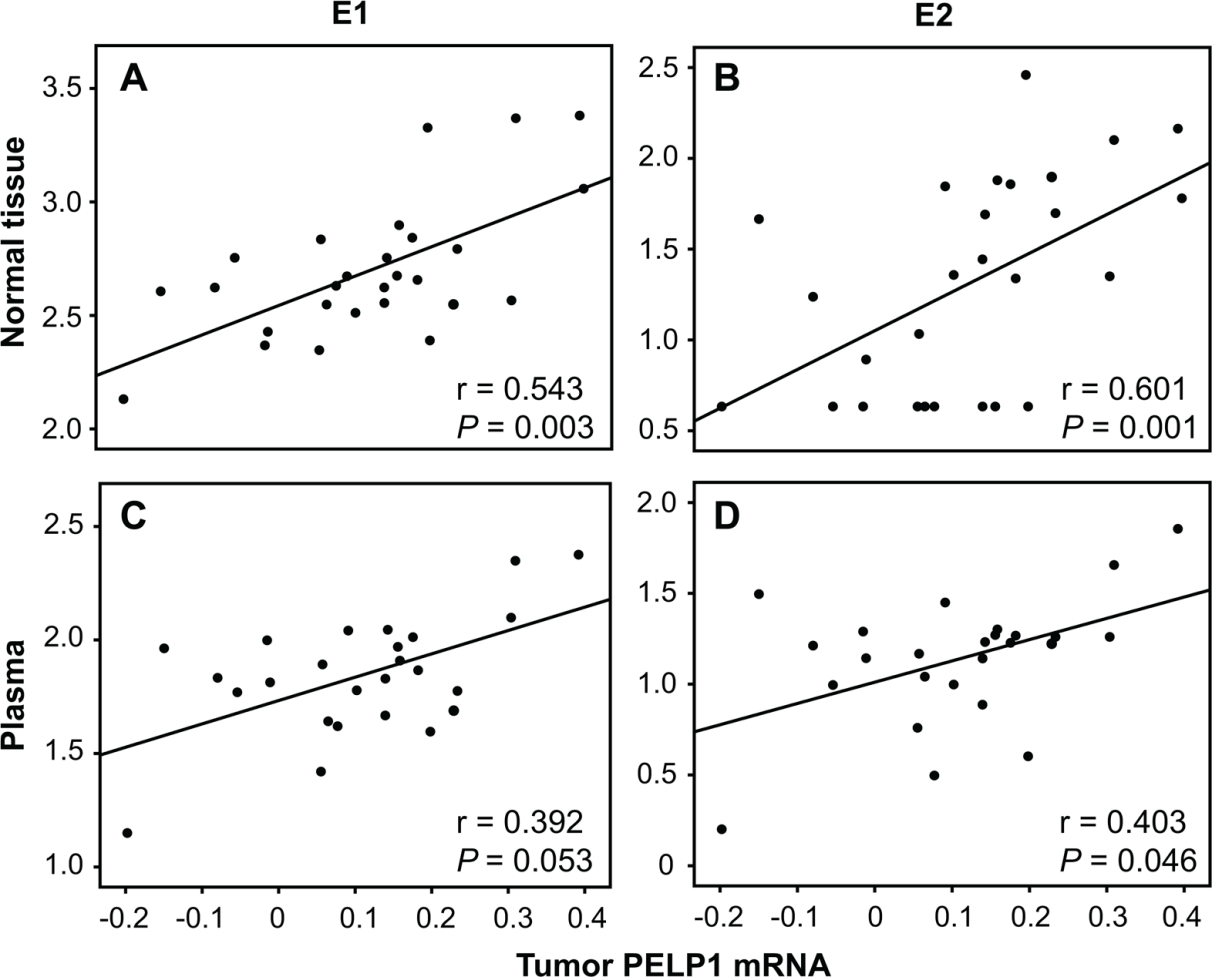
Correlation of *PELP1* tumor levels and estrogens from postmenopausal patients. Scatterplots illustrating correlations of tumor PELP1 mRNA levels with corresponding normal tissue E_1_ (A) and E_2_ (B) levels in addition to E_1_ (C) and E_2_ (D) in plasma in the subgroup of postmenopausal patients. RNA was extracted from tumor tissue and reverse transcriptase (RT)-real-time PCR of *PELP1* and 3 reference genes, *TBP*, *PUM1* and *RPLP0*, was performed in order to analyze relative *PELP1* mRNA tumor levels. Correlations were evaluated using Spearman Rank (two-tailed) test.

In order to elucidate associations between *PELP1* and estrogens *in vivo*, we performed Spearman correlations of tumor *PELP1* mRNA with E_1_, E_2_ and E_1_S levels in tumor, normal breast tissue and plasma from breast cancer patients (Table 2). In all patients, tumor *PELP1* correlated with normal tissue concentrations of E_1_ and E_2_ (r = 0.366, *P* = 0.020 and r = 0.329, *P* = 0.038, respectively). In the postmenopausal subgroup, tumor *PELP1* correlated with E_1_ and E_2_ concentrations in normal tissue (r = 0.543, *P* = 0.003 and r = 0.601, *P* = 0.001, Fig 1A and 1B, respectively) and plasma (r = 0.392, *P* = 0.053 (borderline significance) and r = 0.403, *P* = 0.046, Fig 1C and 1D, respectively). Interestingly, no intratumoral correlations between *PELP1* and estrogens were observed in the total dataset or in the subgroup of postmenopausal patients. Additionally, no correlations at all were observed between tumor *PELP1* levels and estrogens among premenopausal patients (data not shown).

**Table 2:** Correlations of tumor PELP1 mRNA with estrogens in tumor, normal tissue and plasma.

		Tumor PELP1 mRNA
Tumor	E_1_	r = 0.271 (-0.044–0.537, n = 40)
	E_2_	r = 0.160 (-0.159–0.449, n = 40)
	E_1_S	r = 0.151 (-0.168–0.442, n = 40)
Normal tissue	E_1_	r = 0.366 (0.061–0.608, n = 40)*
	E_2_	r = 0.329 (0.019–0.581, n = 40)*
	E_1_S	r = -0.034 (-0.342–0.280, n = 40)
Plasma	E_1_	r = 0.327 (-0.007–0.595, n = 35)
	E_2_	r = 0.313 (-0.023–0.585, n = 35)
	E_1_S	r = 0.268 (-0.072–0.552, n = 35)

Spearman rank correlations (two tailed) with 95% Confidence Intervals

*P ≤ 0.05

**P ≤ 0.01

### Correlations of PELP1 mRNA with estrogens in ER+ tumors

**Fig 2:**
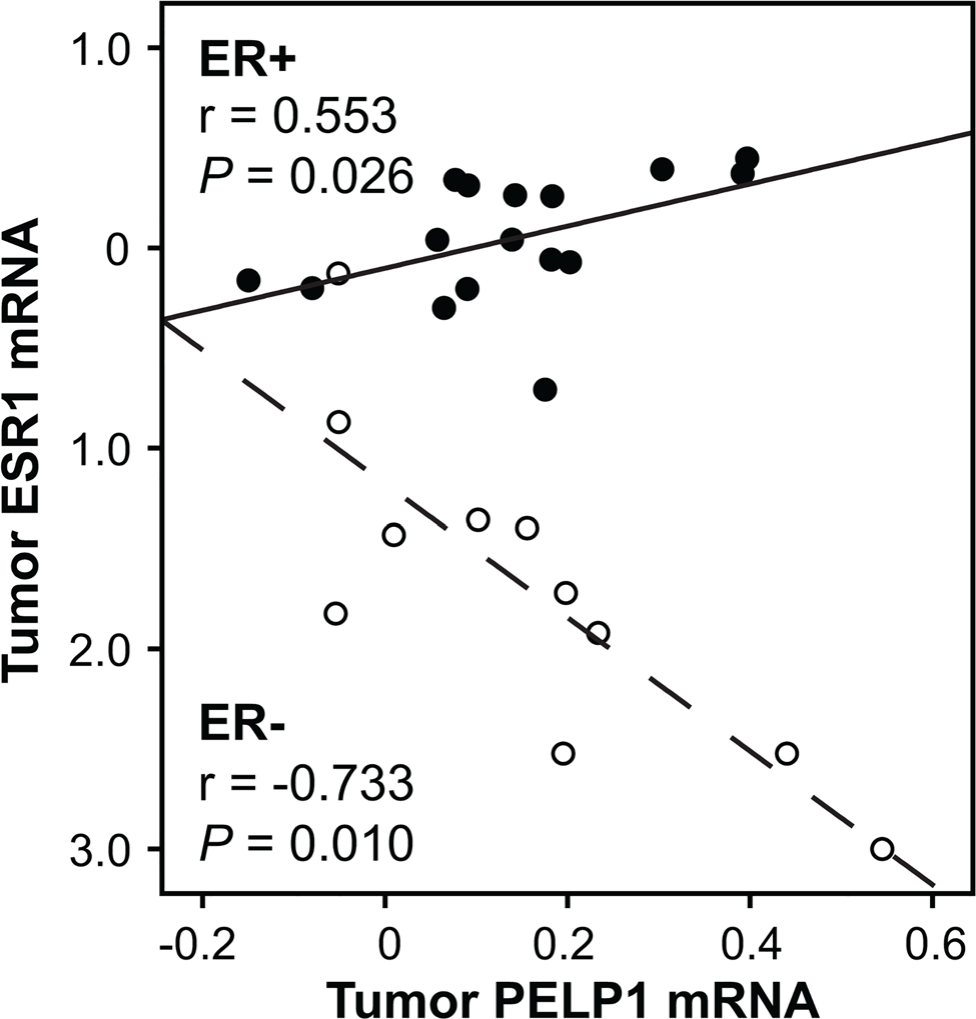
Correlations of *PELP1* with ER mRNA (*ESR1*) levels. Scatterplot illustrating correlations of *PELP1* with *ESR1* in ER- (open circle) and ER+ (filled circle) tumors. Relative *PELP1* expression levels were analyzed using RT- real-time PCR whereas *ESR1* tumor levels are published before [21]. Correlations were evaluated using Spearman Rank (two-tailed) test. Association between *PELP1* and *ESR1* levels when ER status was taken into account was verified using a multivariate logistic regression model (*P* ≤ 0.001).

Further, we conducted analyses in two tumor subgroups, ER+ and ER-, and examined potential correlations of *PELP1* with estrogens (Table 3). Among ER+ patients, *PELP1* tumor levels were positively correlated with tumor tissue E_1_ and E_2_ concentrations (r = 0.562, *P* = 0.003 and r = 0.411, *P* = 0.037, respectively), E_1_ and E_2_ in normal tissue (r = 0.461, *P* = 0.018 and r = 0.427, *P* = 0.030, respectively) and E_1_, E_2_ and E_1_S plasma levels (r = 0.576, *P* = 0.003; r = 0.456, *P* = 0.025 and r = 0.406, *P* = 0.049, respectively). Similar correlations were not observed in the subgroup of ER- tumors (Table 3). Furthermore, *PELP1* levels were positively correlated with *ESR1* in the ER+ tumors (r = 0.553, *P* = 0.026, Fig 2), whereas a negative correlation was observed with *ESR1* in ER- tumors (r = -0.733, *P* = 0.010). In a multivariate model taking ER+/- status into account when assessing the association between *PELP1* and *ESR1* expression, we observed a highly significant correlation (P ≤ 0.001). Taken together, the positive correlations between *PELP1* and estrogen levels in tumor, normal tissue and circulation seem to be exclusive to estrogen responsive ER+ tumors only.

**Table 3:** Correlations between tumor PELP1 mRNA and estrogens in ER- and ER+ breast tumors.

			Tumor PELP1 mRNA
Tumor	E_1_	ER-	r = -0.200 (-0.660–0.370, n = 14)
		ER+	r = 0.562 (0.223–0.780, n = 26)**
	E_2_	ER-	r = 0.073 (-0.476–0.581, n = 14)
		ER+	r = 0.411 (0.028–0.689, n = 26)*
	E_1_S	ER-	r = 0.059 (-0.487–0.572, n = 14)
		ER+	r = 0.182 (-0.221–0.532, n = 26)
Normal tissue	E_1_	ER-	r = 0.196 (-0.373–0.658, n = 14)
		ER+	r = 0.461 (0.090–0.720, n = 26)*
	E_2_	ER-	r = 0.229 (-0.343–0.677, n = 14)
		ER+	r = 0.427 (0.047–0.699, n = 26)*
	E_1_S	ER-	r = -0.091 (-0.462–0.593, n = 14)
		ER+	r = -0.074 (-0.449–0.323, n = 26)
Plasma	E_1_	ER-	r = 0.027 (-0.582–0.617, n = 11)
		ER+	r = 0.576 (0.225–0.795, n = 24)**
	E_2_	ER-	r = 0.109 (-0.525–0.665, n = 11)
		ER+	r = 0.456 (0.064–0.726, n = 24)*
	E_1_S	ER-	r = 0.209 (-0.449–0.719, n = 11)
		ER+	r = 0.406 (0.003–0.695, n = 24)*

Abbreviations: ER, estrogen receptor; E_1_, estrone; E_2_, estradiol; E_1_S, estrone sulphate

Spearman rank correlation (two tailed) with 95% Confidence Intervals

*P ≤ 0.05

**P ≤ 0.01

We further restricted our analysis to postmenopausal women with ER+ disease, showing that *PELP1* tumor levels were positively correlated with E_1_ concentration in tumor (r = 0.583, *P* = 0.009, Table 4), E_1_ and E_2_ concentration in normal tissue (r = 0.679, *P* = 0.001; r = 0.618, *P* = 0.005, respectively) and E_1_ and E_2_ plasma concentrations (r = 0.653, *P* = 0.003; r = 0.519, *P* = 0.027, respectively). Additionally, *PELP1* and *ERS1* intratumoral expression levels were positively correlated in ER+ tumors from postmenopausal women (r = 0.593, *P* = 0.033).

**Table 4:** Correlations between *PELP1* and estrogens in ER+ tumors from postmenopausal patients.

		Tumor PELP1 mRNA
Tumor	E_1_	r = 0.583 (0.175–0.820, n = 19)**
	E_2_	r = 0.444 (-0.013–0.747, n = 19)
	E_1_S	r = 0.131 (-0.344–0.552, n = 19)
Normal tissue	E_1_	r = 0.679 (0.323–0.866, n = 19)**
	E_2_	r = 0.618 (0.228–0.837, n = 19)**
	E_1_S	r = -0.332 (-0.144–0.683, n = 19)
Plasma	E_1_	r = 0.653 (0.268–0.858, n = 18)**
	E_2_	r = 0.519 (0.069–0.794, n = 18)*
	E_1_S	r = 0.408 (-0.073–0.735, n = 18)
*ERS1* tumor		r = 0.593 (0.062–0.862, n = 13)*

Abbreviations: ER, estrogen receptor; E_1_, estrone; E_2_, estradiol; E_1_S, estrone sulphate; ERS1, estrogen receptor mRNA

Spearman rank correlation (two tailed) with 95% Confidence Intervals

*P ≤ 0.05

**P ≤ 0.01

### PELP1 mRNA is negatively correlated with EGFR1/HER1 and NRG1 mRNA

**Fig 3:**
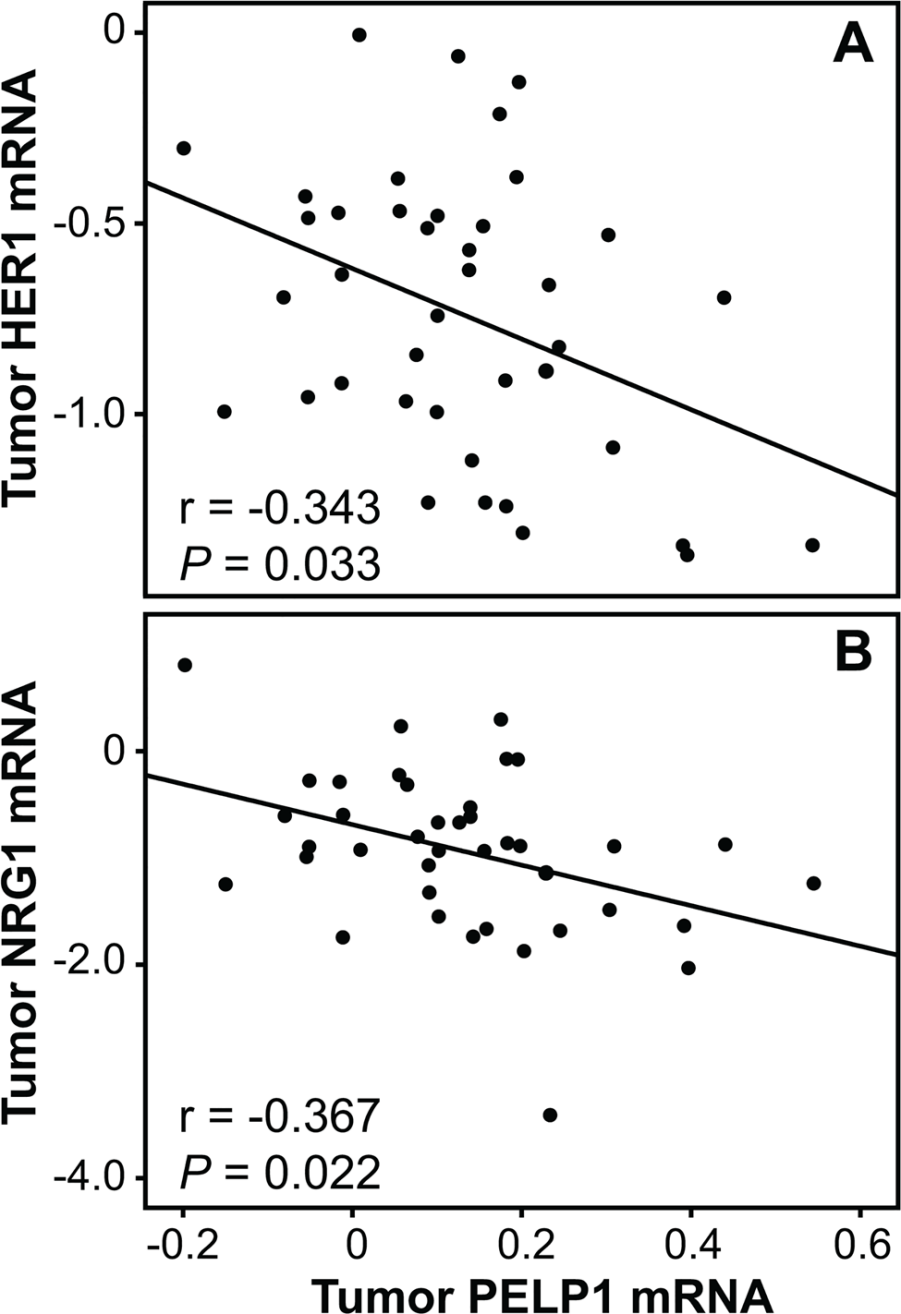
Intratumoral correlations of *PELP1* with *HER1* and *NRG1*. Tumor *PELP1* mRNA levels were correlated with *HER1* (A) and *NRG1* (B) tumor levels. Relative *PELP1* expression levels were analyzed using RT- real-time PCR whereas *HER1* and *NRG1* tumor levels are published before [20]. Correlations were evaluated using Spearman Rank (two-tailed) test.

Previously, we have demonstrated that the mRNA expression levels of epidermal growth factor receptor (EGFR or HER1) and the ligand and growth factor Neuregulin (NRG1) are negatively regulated by estrogens in this data set [20]. Thus, we analyzed any correlations between *PELP1*, *HER1* and *NRG1*. We observed *PELP1* to be negatively correlated with both *HER1* (Fig 3; r = -0.343, *P* = 0.033) and *NRG1* (r = -0.367, *P* = 0.022) within tumor tissue. Taken together, these results support the theory that *PELP1* is positively regulated by estrogens *in vivo*.

## Discussion

It is well known that estrogens and ER regulate breast cancer cell proliferation [1]. However, ER signaling is complex and involves multiple coregulatory proteins [24] including PELP1, an ER coactivator that is dysregulated in breast cancer [6, 9, 10]. In this clinical study, *PELP1* mRNA expression levels in matched malignant and normal breast tissue from breast cancer patients were analyzed and correlated with E_1_, E_2_ and E_1_S levels. Our results suggest *PELP1* to be associated and potentially regulated by estrogens in ER+ breast cancers.

PELP1 has previously been shown to be overexpressed in 60–80% of breast tumors [6, 9]. In line with this, we found *PELP1* levels to be higher in malignant- as compared to normal breast tissue, in our dataset. High PELP1 protein expression has been associated with tumor grade, proliferation, node-positive invasive cancer and distant metastases, decreased breast cancer specific survival and disease free survival [6–8]. Recent data suggest that PELP1 promotes oncogenesis by alternative splicing, leading to the activation of unique pathways. This occurs in addition to modulation of epigenetic alternations at ER target promoters [12, 14, 25, 26].

PELP1 is known to be modulated by the E_2_-ER pathway [17]. However, to the best of our knowledge, no associations between PELP1 and *in vivo* breast cancer estrogen levels have been shown previously. The finding that *PELP1* tumor levels correlated with normal tissue estrogens indicates an association between PELP1 and estrogens in breast cancer patients. Considering the fact that tissue- and plasma estrogen levels are elevated in pre- compared to postmenopausal women [19], we analyzed these subgroups separately. We found *PELP1* tumor levels in postmenopausal patients to be positively associated with estrogen levels in normal tissue and plasma. Although, the presence of association cannot establish causality, this may indicate that tumor *PELP1* is regulated by estrogen in these patients. Cyclic secretion of hormones among premenopausal women during menstrual cycle complicates the analyses of associations between plasma estrogens and other biomarkers and may explain the lack of correlations between *PELP1* and estrogens in either tissue or serum.

PELP1 is an estrogen-regulated ER target gene, exhibiting proximal estrogen responsive element (ERE) half sites in its promoter [17]. In addition, PELP1 may regulate aromatase and, thereby, modulate *in situ* estrogen synthesis in tumors [27]. Since E_2_ levels are markedly higher in ER+ compared to ER- tumors [19], we explored associations between *PELP1* in these two tumors separately. *PELP1* in ER+ tumors was found to be associated with E_1_ and E_2_ concentrations in plasma, normal tissue and tumors. In contrast, no associations between *PELP1* and estrogens were observed within ER- tumors. Relationships between plasma estrogen levels and expression of estrogen-dependent genes in ER+ tumors have also been shown by others [28], underlining the fact that tissue and plasma estrogen levels in general are at equilibrium [29]. The increase of estrogen levels in breast tumors as compared to surrounding tissues has been suggested to be a consequence of enhanced uptake of estrogens from the circulation and binding of estrogens to ER, rather than *in situ* estrogen synthesis in the tumor [21, 29]. If the association between *PELP1* and estrogens levels was a consequence of increased *in situ* estrogen synthesis mediated by PELP1, we would expect no correlations between *PELP1* in tumors and normal tissue and serum estrogens. Thus, based on these data, and the knowledge that *PELP1* is an estrogen regulated gene [17], we hypothesize that the tumor *PELP1* level is regulated by circulating estrogens. Moreover, we have previously reported the epidermal growth factor receptor *HER1* and the growth factor *NRG1*, to be suppressed by estrogens in tumors [20]. The negative correlation between *PELP1* and *HER1* and *NRG1* observed in this data set further support our hypothesis that *PELP1* is positively regulated by estrogens *in vivo*.

PELP1 expression has been suggested to play a significant role in both ER+ and ER- breast cancer [6, 7, 9]. Our finding that *PELP1* was positively correlated with *ESR1* in ER+ tumors and negatively correlated with *ESR1* in ER- tumors supports the theory that PELP1 may have differential roles in these tumors. PELP1 is a unique protein exhibiting ten nuclear receptor interacting boxes (LXXLL motifs) important for interacting with ER, additionally to several consensus PXXP motifs facilitating interaction with proteins containing SH3 domains [10, 11]. Due to these interacting domains and its presence both in the nucleus and cytoplasm, PELP1 is involved in multiple biological processes that may have different effects in ER+ and ER- tumors.

The present study has some limitations. The sample size is small, especially when subgroups of patients based on ER-status or menopausal stage are analyzed. Previous data from *in vitro* studies support our findings showing a positive correlation between estrogens and *PELP1* [17], but we cannot exclude other unknown factors that may have regulatory effects on *PELP1 in vivo*, and the results should be interpreted with caution. Another limitation of our study is that only mRNA and not protein levels have been analyzed. It should be noted that changes in mRNA and protein expression does not always go in parallel, and other regulatory mechanisms such as posttranslational modifications or changes in turnover may affect the protein expression of PELP1. Thus, in another clinical study, associations between estrogens and PELP1 should be confirmed on the protein level.

PELP1 is a transcriptional regulator recruiting other coregulators and remodeling chromatin to facilitate access to the promoter of its target genes [30]. Recent results based on RNA-sequencing have demonstrated that PELP1 regulates a number of genes involved in estrogen signaling, breast cancer progression and RNA splicing [26]. Moreover, PELP1 deregulation alters expression of ER-targets genes *in vivo* [18]. PELP1-driven tumors are known to be ER+ and have excessive activation of Src and MAPK [31]. PELP1 interacts with Src and acts as a scaffold protein, mediating ER-Src interaction [11, 32]. The ER-Src axis may promote hormonal resistance by proto-oncogenes such as PELP1 and HER2 [31]. The present data, revealing associations between *PELP1*and estrogens in ER+ tumors, contribute to the accumulating evidence of PELP1’s tumorigenic behavior.

## Conclusions

In summary, this study provides novel information regarding the association of the ER-coactivator PELP1 with estrogens in breast cancer patients. We hypothesize that estrogens influence PELP1 mRNA expression in breast tumor tissue, suggesting a potential important role of PELP1 in ER+ breast cancer growth *in vivo*.

## Supporting Information

S1 Fig *PELP1* in normal and tumor tissue. (EPS)

S2 Fig Tumor *PELP1* levels in ER+ and ER- tumors and from pre and postmenopausal subjects. (EPS)

S1 Table Primer and probes for real-time PCR. (DOCX)
