# Supplementary material for: Estrogens Correlate with PELP1 Expression in ER Positive Breast Cancer
Source: PLoS One. 2015 Aug 6;10(8):e0134351. doi: 10.1371/journal.pone.0134351 (PMC4527840; doi:10.1371/journal.pone.0134351)
Supplement: S1 Table — (DOCX) [file pone.0134351.s003.docx]

| Gene | Forward primer | Reverse primer | UPL Probe |
| --- | --- | --- | --- |
| PELP1 | ggatagcaatgccaacagc^a^ | gaggcccacacatgagga | 30 |
| PUM1 | tcacatggatcctcttcaagc | cctggagcagcagagatgtat | 86 |
| RPLP0 | tctacaaccctgaagtgcttgat | caatctgcagacagacactgg | 6 |
| TBP^b^ |  |  |  |

**Table S1. Primer and probes for real-time PCR.**

^a^ Primers and probes were designed using Universal Probe Library (Roche, Basel, Switzerland)

^b^ A reference analysis kit was used for the TATA-box binding protein (TBP) reference gene (Roche)
